# Non-Equilibrium Thermodynamics and Stochastic Dynamics of a Bistable Catalytic Surface Reaction

**DOI:** 10.3390/e20110811

**Published:** 2018-10-23

**Authors:** Miguel Pineda, Michail Stamatakis

**Affiliations:** Department of Chemical Engineering, University College London, Roberts Building, Torrington Place, London WC1E 7JE, UK

**Keywords:** fluctuations, chemical master equation, bistability, entropy production rate, non-equilibrium steady state

## Abstract

Catalytic surface reaction networks exhibit nonlinear dissipative phenomena, such as bistability. Macroscopic rate law descriptions predict that the reaction system resides on one of the two steady-state branches of the bistable region for an indefinite period of time. However, the smaller the catalytic surface, the greater the influence of coverage fluctuations, given that their amplitude normally scales as the square root of the system size. Thus, one can observe fluctuation-induced transitions between the steady-states. In this work, a model for the bistable catalytic CO oxidation on small surfaces is studied. After a brief introduction of the average stochastic modelling framework and its corresponding deterministic limit, we discuss the non-equilibrium conditions necessary for bistability. The entropy production rate, an important thermodynamic quantity measuring dissipation in a system, is compared across the two approaches. We conclude that, in our catalytic model, the most favorable non-equilibrium steady state is not necessary the state with the maximum or minimum entropy production rate.

## 1. Introduction

The study of dissipative systems has been an active research topic during many years, and a plethora of interesting studies have been published [1]. Nowadays, the notion of dissipative structures appears in a wide range of fields from physics to the economics and social sciences [2]. These dissipative structures, which are characterised by the existence of non-equilibrium steady states, are created by irreversible processes that dissipate energy and generate entropy [1,2]. However, a question of current interest is whether the entropy production rate reaches an extremum (maximum/minimum) or not at the non-equilibrium steady state (NESS) [3,4,5,6,7,8,9,10,11,12,13,14]. In this work, we explore this problem from the viewpoint of bistable catalytic reaction networks.

The idea that universal extremal principles determine many of the phenomena that occur in nature has been around for many years. The point is that, independent of the form of the specific system, among all possible trajectories that may link an initial state from the final one, the trajectory that will be actually followed, under the laws governing the system, extremizes a certain quantity. One of the most famous extremal principle is the principle of least action for conservative systems, which is used to derive many physical theories, like for example Maxwell’s equations, Newton’s laws, and quantum mechanics [15]. The extend to which this type of principles can be expected in systems operating far from the state of thermodynamic equilibrium (dissipative systems) has attracted interest during many decades. However, no general principles of this kind have been rigorously proven, and many conflicting results exist [3,4,5,6,7,8,9,10,11,12,13,14]. A well known extremal principle is the so-called Prigogine’s minimum entropy production principle (MinEPP) which states that, through appropriate constraints and close to equilibrium where the fluxes can be expressed as a linear function of the thermodynamic forces, the NESS of a system is that state in which the entropy production rate has the minimum value [1]. According to this MinEPP, a fluctuation from the NESS can only increase the entropy production rate above the stationary value. Then, irreversible processes drive back the entropy production to its minimum value at the NESS. In this sense, MinEPP compares steady states with transients or non-steady states. Another interesting extremal principle that has also emerged is the maximum entropy production principle (MaxEPP) of Paltridge and others [9,12,16,17,18,19]. This MaxEPP states that, for systems far from equilibrium that admit multiple non-equilibrium steady states, the system is most likely to be found in the NESS with the greatest entropy production [3,4,13,16]. In this sense, the MaxEPP compares non-equilibrium steady states to other non-equilibrium steady states. It is also important to mention that the state of thermodynamic equilibrium can be characterised either by the principle of maximum entropy or the principle of the minimum (in this case equal to zero) entropy production [1]. It is this latter principle that naturally extends to the linear regime, close to equilibrium. However, far from thermodynamic equilibrium the situation is more complex and conclusive answers regarding universal extremal principles do not exist.

Catalytic surface reactions are typical examples of chemical systems operating far-from-equilibrium which develop dissipative structures, exhibiting for instance bistability [20,21]. In these systems, a planar crystalline surface is exposed to a gas mixture of reactants and the distance from equilibrium is controlled by the ratio of reactant-to-product partial pressures. At the macroscale, the phenomenon of bistability has been studied both theoretically and experimentally [22,23]. Based on knowledge about the individual steps forming the reaction mechanisms, this phenomenon has been successfully modelled by the solution of sets of ordinary differential equations (ODEs) for the variables describing the surface concentrations of the species involved. These ODEs predict that, once the system reaches the NESS exhibited by the governing equations, it will remain there for an indefinite period of time. However, at the mesoscale, molecular fluctuations become important and randomly affect the reaction kinetics of the catalytic system [24,25,26]. This is the case for the catalytic CO oxidation on Pt field-emitter-tips and on oxide-supported Pd nanoparticles, where the phenomenon of fluctuation-induced transitions between the two NESS branches of the bistable region has been observed experimentally [25,26]. The experimental observations have been rationalised by the theory of stochastic process [27,28]. These findings make the bistable CO oxidation on surfaces (an important step in automotive exhaust catalysis [29]) a suitable and interesting framework to study entropy production rate in systems with multiple non-equilibrium steady states.

In this work, with an interest in the deterministic and stochastic behaviour of catalytic surface reaction networks, we focus on a minimalistic model for the bistable catalytic CO oxidation on well-defined crystal surfaces and investigate trends in the entropy production rate (the basic thermodynamic quantity measuring dissipation) when the catalytic system is at a NESS. We address this question from the deterministic and stochastic viewpoint. Our simulation results suggest that, for our bistable catalytic system, the tendency to minimise or maximise dissipation does not reflect a universal trend but is, rather, control parameter and initial condition specific.

The paper is organised as follows. In Section 2, we introduce the bistable catalytic reaction model. Then, in Section 3 we introduce the stochastic and deterministic expression for the entropy production rate to be implemented in this work. We continue with the presentation and discussion of the results in Section 4. The summary and conclusions are presented in Section 6.

## 2. The Bistable Catalytic Reaction Model

The elementary steps of CO oxidation on metal surfaces are determined by the so-called Langmuir-Hinshelwood (LH) mechanism consisting of the following steps

(1) CO(gas)+∗⇄CO(ads),

(2) $$O_{2}\left(\mathrm{gas}\right)+2*⇄2O(\mathrm{ads}),$$

(3) $${\mathrm{CO}}_{2}\left(\mathrm{gas}\right)+2*⇄\mathrm{CO}(\mathrm{ads})+O(\mathrm{ads}).$$

with ∗ and ads denoting a vacant site on the surface and adsorbed atoms or molecules, respectively [30]. For thermodynamic consistency, each forward reaction is accompanied by the corresponding reverse reaction. On surfaces with high adsorbate motility, the catalytic system is well mixed and the phenomenon of bistability is one of its most prominent non-equilibrium features. Thus, two different stable non-equilibrium steady states coexist for the same control parameter values (we refer to Table 1 for a description of the control parameters of the model). The less active NESS where, a high CO coverage inhibits the dissociative O${}_{2}$ adsorption, and the active NESS, where the surface is predominantly oxygen covered. The active (less active) NESS is characterised by a high (low) CO${}_{2}$ production rate. [22,23].

In the so-called mean-field approach, where spatial correlations are ignored, the bistable catalytic system is mathematically described with ODEs for the coverage of chemical species involved. This approach predicts that, depending on the initial conditions, the system will reside on one of the two non-equilibrium steady states for an indefinite period of time [23]. However, this prediction breaks down when the surface area is of meso or nanoscale dimensions. By decreasing the area of the catalytic surface, random coverage fluctuations become important and transitions, between the two non-equilibrium steady states, occur [25,26,27,28]. Thus, in order to take into account these random aspects, a stochastic mean-field description is needed. Such a stochastic approach and its macroscopic deterministic limit are discussed in the following sections.

## 3. Theoretical Framework

In this section, we present the chemical master equation (CME) of the catalytic reaction model, valid in the limit of very fast diffusion and Markovian dynamics [31,32]. We also introduce the average stochastic expression for the entropy production rate. From the stochastic description, we derive a set of ODEs for the CO and oxygen coverages on the surface. The associated macroscopic entropy production rate is also presented. In the next section, we explore whether the entropy production is maximised, minimised, or achieves no extremum in the non-equilibrium steady states.

### 3.1. Mean-Field Stochastic Description

We assume that the catalytic system is described by a continuous-time Markov process defined on a discrete space of states given by the vector $Z=\{N_{CO},N_{O}\}$, where $N_{CO}$ is the number of adsorbed CO species on the surface, and $N_{O}$ is the number of adsorbed oxygen atoms. According to Equations (1)–(3), we have three forward reactions (σ=+1, +2, +3) and three reverse reactions (σ=−1, −2, −3). The stoichiometric vectors for forward reactions are $\omega_{+1}=\left\{1,0\right\}$ (for CO(gas) adsorption), $\omega_{+2}=\left\{0,2\right\}$ (for O${}_{2}$(gas) dissociative adsorption), and $\omega_{+3}=\left\{1,1\right\}$ (for CO${}_{2}$(gas) dissociative adsorption). The corresponding vectors for the reverse reactions are $\omega_{-1}=-\omega_{+1}$ (for CO(ads) desorption), $\omega_{-2}=-\omega_{+2}$ (for associative O(ads) desorption), and $\omega_{-3}=-\omega_{+3}$ (for CO(ads) + O(ads) reaction). The maximum allowed number of particles on the surface is $N_{L}$. Because a reverse reaction is associated with each forward reaction, an elementary event of the reaction σ∈{±1,±2,±3} induces the random jump

(4) $$Z\overset{\sigma }{\to }Z+\omega_{\sigma }$$

with the transition rate $W_{\sigma }\left(Z\to Z+\omega_{\sigma }\right)$. Similarly, an elementary event of the reverse reaction -σ induces the random jump

(5) $$Z\overset{-\sigma }{\to }Z+\omega_{-\sigma },$$

with the transition rate $W_{-\sigma }\left(Z\to Z+\omega_{-\sigma }\right)$.

The random jumps and waiting times are normally obtained using the so-called Gillespie algorithm [24,33]. When the system is in state Z, the next reaction σ to occur is a random variable with probability given by

(6) $$P_{\sigma }=\frac{W_{\sigma }\left(Z\to Z+\omega_{\sigma }\right)}{\sum_{\sigma^{{}^{\prime }}}^{}W_{\sigma^{{}^{\prime }}}\left(Z\to Z+\omega_{\sigma^{{}^{\prime }}}\right)},$$

while the waiting time τ is also a new random variable which is exponentially distributed with the probability density.

(7) p(τ)=kexp(−kτ),

where

(8) $$k=\sum_{\sigma^{{}^{\prime }}}^{}W_{\sigma^{{}^{\prime }}}\left(Z\to Z+\omega_{\sigma^{{}^{\prime }}}\right).$$

Please note that the successive jumps are statistically independent. In Equations (6) and (8), the sum over the reactions runs over both the forward and reverse reactions $\sigma^{{}^{\prime }}=\pm 1,\pm 2,\pm 3$.

The vector Z follows as stochastic trajectory, and therefore, it is characterised by the probability P(Z;t) of having a number Z of particles on the surface at time *t*. The temporal evolution of this probability distribution is given by the following CME

(9) $$\frac{d}{dt}P\left(Z;t\right)=\sum_{\sigma }^{}\left[W_{\sigma }\left(Z-\omega_{\sigma }\to Z\right)P\left(Z-\omega_{\sigma };t\right)-W_{-\sigma }\left(Z\to Z+\omega_{-\sigma }\right)P\left(Z;t\right)\right],$$

where the sum runs over both the forward and reverse reactions (for this notation see [34,35]). The terms $W_{\sigma }$ stand for the transition rates for the forward and reverse reactions (see Table 1 and Appendix). The validity of the Markovian approximation is based on the idea that there exists a clear separation of time scales between the dynamics of the stochastic observables and that of the faster (microscopic) degrees of freedom that are not included in the stochastic description. Because of this time scale separation, the microscopic degrees of freedom follow an equilibrium distribution all along the stochastic trajectories.

The probability distribution relaxes toward a stationary state value, P(Z;t) = $P_{st}\left(Z\right)$, characterised by a NESS or the state of thermodynamic equilibrium. If the stationary state becomes the equilibrium one ($P_{st}\left(Z\right)=P_{eq}\left(Z\right)$), the detailed balance (for all the reactions σ∈{±1,±2,±3})

(10) $$W_{\sigma }\left(Z-\omega_{\sigma }\to Z\right)P_{eq}\left(Z-\omega_{\sigma }\right)=W_{-\sigma }\left(Z\to Z+\omega_{-\sigma }\right)P_{eq}\left(Z\right),$$

applies. In equilibrium, the solution of Equation (9) is a multinomial distribution (see Appendix A). For a NESS, detailed balance does not hold and an analytical expression for $P_{st}\left(Z;t\right)$ is not available. However, in the following, the steady state probability distribution will be obtained after averaging over individual trajectories generated by the Gillespie stochastic simulation algorithm. In the next section, we introduce the corresponding stochastic entropy production rate.

#### Stochastic Entropy Production Rate

The most important thermodynamic quantity measuring dissipation is the so-called entropy production rate. The entropy of the Markov process described by the probability distribution P(Z;t) is given by

(11) $$S\left(t\right)=-\sum_{Z}^{}P\left(Z;t\right)\ln P(Z;t)=\langle -\ln P(Z;t)\rangle ,$$

in units where the Boltzmann constant is equal to one, $k_{B}=1$ [34,36,37,38]. Equation (11) is simply a contribution to entropy due to the probability distribution of the number of CO molecules and oxygen atoms on the surface. It is assumed to be valid in equilibrium and as well as in non-equilibrium situations [34,36,37,38]. The sum over the vector Z means a double sum over $N_{CO}$ and $N_{O}$.

The time evolution of Equation (11) can be written as [34,36,37,38]

(12) $$\frac{dS}{dt}=\frac{1}{2}\sum_{Z,\sigma }^{}J_{\sigma }\left(Z;t\right)\ln\frac{P(Z-\omega_{\sigma };t)}{P(Z;t)},$$

where we define the net reaction rates or mesoscopic thermodynamic fluxes from state $Z-\omega_{\sigma }$ to Z as

(13) $$J_{\sigma }\left(Z;t\right)=W_{\sigma }\left(Z-\omega_{\sigma }\to Z\right)P\left(Z-\omega_{\sigma };t\right)-W_{-\sigma }\left(Z\to Z+\omega_{-\sigma }\right)P\left(Z;t\right).$$

As above, in Equation (12), the sum over the reactions runs over both the forward and reversed reactions σ=±1,±2,±3.

Equation (12) represents the total variation of entropy, which is also obtained from

(14) $$\frac{dS_{i}}{dt}=\frac{dS}{dt}-\frac{dS_{e}}{dt},$$

where $\frac{dS_{i}}{dt}$ is the entropy production rate due to internal processes and $\frac{dS_{e}}{dt}$ is the net entropy flow rate, which can be positive or negative [1]. This net entropy flow rate is calculated from the exchange fluxes of entropy into and out of the system. The entropy production should meet two conditions: it should be non-negative and vanish in equilibrium. An expression that satisfies these requirements is provided by Schnakenberg [39], which relates the entropy production rate to the transition rates of the master equation as

(15) $$\frac{dS_{i}}{dt}=\frac{1}{2}\sum_{Z,\sigma }^{}J_{\sigma }\left(Z;t\right)A_{\sigma }\left(Z;t\right),$$

where

(16) $$A_{\sigma }\left(Z;t\right)=\ln\frac{W_{\sigma }\left(Z-\omega_{\sigma }\to Z\right)P\left(Z-\omega_{\sigma };t\right)}{W_{-\sigma }\left(Z\to Z+\omega_{-\sigma }\right)P\left(Z;t\right)},$$

are the so-called affinities or mesoscopic thermodynamic forces associated with the reactions σ=±1,±2,±3. Because the inequality $\left(R_{\sigma }-R_{-\sigma }\right)\ln R_{\sigma }/R_{-\sigma }\geq 0$, the entropy production rate is always positive in agreement with the second law of thermodynamics [34,36,37,38]. Please note that $(R_{\sigma }-R_{-\sigma })>0$ implies $\ln R_{\sigma }/R_{-\sigma }>0$ and $(R_{\sigma }-R_{-\sigma })<0$ implies $\ln R_{\sigma }/R_{-\sigma }<0$.

The entropy flow is obtained by replacing Equation (12) and Equation (15) into Equation (14). The result is

(17) $$\frac{dS_{e}}{dt}=-\frac{1}{2}\sum_{Z,\sigma }^{}J_{\sigma }\left(Z;t\right)\ln\frac{W_{\sigma }\left(Z-\omega_{\sigma }\to Z\right)}{W_{-\sigma }\left(Z\to Z+\omega_{-\sigma }\right)}$$

The equations presented above define the ensemble stochastic thermodynamics of our catalytic system for equilibrium and non-equilibrium conditions [40]. In a NESS, dS/dt = 0, implying

(18) $$\frac{dS_{e}}{dt}=-\frac{dS_{i}}{dt},$$

which means that the net entropy exchanged with the environment must be negative. Please note that the entropy flow is easier to calculate and can be used in place of the stochastic entropy production rate, when dealing with non-equilibrium steady states. As expected, at thermodynamic equilibrium, the entropy flow and entropy production are zero.

After having presented the stochastic framework to be implemented in this work, we proceed in the following section to discuss the deterministic limit of it.

### 3.2. Deterministic Mean-Field Description

In the macroscopic limit, the dynamics of the CO coverage (*u*) and the oxygen coverage (ν) are described by the following two coupled first-order nonlinear ODEs

(19) $$\frac{du}{dt}=w_{+1}-w_{-1}+w_{+3}-w_{-3},$$

(20) $$\frac{d\nu }{dt}=w_{+2}-w_{-2}+w_{+3}-w_{-3},$$

which are obtained from the so-called chemical Fokker-Planck equation (CFPE) approximation of the CME, in the limit of $N_{L}\to \infty$. This CFPE is obtained after truncating the Kramers-Moyal expansion of the CME (Equation (9)) [27,41,42]. The forward and reverse reaction rates or fluxes are given in Table 2. At steady state, where du/dt = dν/dt=0, there can be one, two or three non-negative steady state solutions of the system of equations. A solution corresponds to certain coverage of CO and oxygen on the surface. The macroscopic thermodynamic equilibrium condition, where each forward reaction rate is equated to its corresponding reverse reaction ($w_{\sigma }=w_{-\sigma }$, for all the reactions σ∈{±1,±2,±3}), is characterised by one single solution (the equilibrium one). This macroscopic thermodynamic equilibrium condition leads to [7]

(21) $$\left(\frac{K_{co}^{ads}}{K_{co}^{des}}\right){\left(\frac{K_{o_{2}}^{ads}}{K_{o}^{des}}\right)}^{1/2}\left(\frac{K_{r}}{K_{co_{2}}}\right)=1,$$

as expected. However, far from equilibrium up to three non-negative steady state solutions can exist. Here, we are interested in the bistable case where two non-equilibrium steady states are separated by an unstable one. However, first, let us introduce the associated macroscopic entropy production rate of the catalytic system.

### 3.3. Macroscopic Entropy Production Rate

In the macroscopic limit (i.e., $N_{L}\to \infty$), the entropy production rate to maintain the NESS is obtained from Equations (17) and (18). It is given by the following sum

(22) $$EP=\frac{1}{2}\sum_{\sigma }^{}\left(w_{+\sigma }-w_{-\sigma }\right)\ln\frac{w_{+\sigma }}{w_{-\sigma }}\geq 0,$$

in units in which $k_{B}$ = 1 [34]. We refer to [1,40] for a derivation of this expression. However, note that Equation (22) should be evaluated at the corresponding steady state value. The sum over the reactions runs over both the forward and reverse reactions σ=±1,±2,±3. Each term is the product of the net reaction rate or macroscopic thermodynamic flux ($w_{+\sigma }-w_{-\sigma }$) and the macroscopic thermodynamic force or affinity ($\ln\frac{w_{+\sigma }}{w_{-\sigma }}$) [34]. At thermodynamic equilibrium, there is no net entropy production rate because the thermodynamic fluxes and forces vanish. Because of the inequality $\left(w_{+\sigma }-w_{-\sigma }\right)\ln w_{+\sigma }/w_{-\sigma }\geq 0$, the macroscopic entropy production rate is always positive in agreement with the second law of thermodynamics. It is also important to mention that, in the limit $N_{L}\to \infty$, $EP=N_{L}^{-1}dS_{i}/dt$ [43]. Please note that this last statement follows from Equation (18).

## 4. Results and Discussion

Let us start with the deterministic analysis of the catalytic system. Then, we continue with the corresponding stochastic framework. It is known from experiments that, in the range where bistability exists, the oxygen coverage is small [23]. Therefore, for all cases, $k_{o_{2}}^{ads}=0.2$, $k_{o}^{des}=20$, $k_{co_{2}}=0.5$, $k_{r}=50$, and ζ=4 (square lattice). The system of ODEs is solved by using the so-called Euler method and the probability distribution for the stochastic description is constructed from stochastic trajectories generated by Gillespie’s algorithm together with the transition rates presented in Table 1.

### 4.1. Deterministic Analysis

In the macroscopic regime, where the deterministic approach is valid, the condition of thermodynamic equilibrium leads to the following equilibrium relations

(23) $$\nu_{eq}=\frac{1-u_{eq}}{1+\sqrt{k_{o}^{des}/k_{o_{2}}^{ads}}},$$

(24) $${\left(1-u_{eq}-\nu_{eq}\right)}^{2}-\frac{2k_{r}u_{eq}\nu_{eq}}{k_{co_{2}}}=0,$$

and

(25) $$k_{co}^{ads}=\left(\frac{u_{eq}}{1-u_{eq}-\nu_{eq}}\right)k_{co}^{des},$$

as expected.

This allows us to determine the equilibrium or thermodynamic branch in the parameter space ($k_{co}^{ads}$, $k_{co}^{des}$). Note also that, from Equation (21), one gets

(26) $$k_{co}^{ads}=\left[\frac{k_{co_{2}}}{2k_{r}}{\left(\frac{k_{o}^{des}}{k_{o_{2}}^{ads}}\right)}^{1/2}\right]k_{co}^{des}.$$

**Figure 1:**
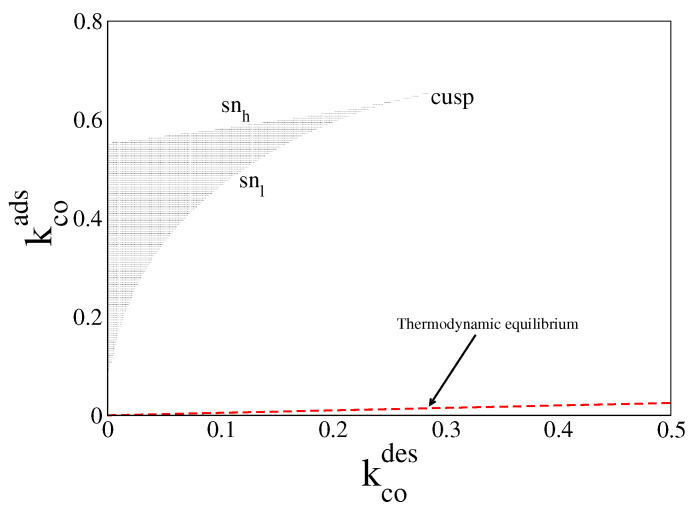
Steady state bifurcation diagram in the parameter space ($k_{co}^{ads}$, $k_{co}^{des}$) from Equations (19) and (20). Inside black region the system exhibits the bistable phenomenon. Its boundaries are given by the upper saddle node ($sn_{h}$) line and the lower saddle node ($sn_{l}$) line meeting each other in a cusp. Along the dashed-red line ($k_{co}^{ads}\approx 0.05k_{co}^{des}$), the system relaxes to thermodynamic equilibrium. Other parameters are $k_{o_{2}}^{ads}=0.2$, $k_{o}^{des}=20$, $k_{co_{2}}=0.5$, $k_{r}=50$, and ζ=4 (square lattice).

In Figure 1, we plot in red dashed line the equilibrium line given by Equation (25), with $u_{eq}$ and $\nu_{eq}$ obtained from Equations (23) and (24) (or Equation (26)). For parameter values along this line, the system always relaxes to the equilibrium steady state, ($u_{eq}\approx 0.044$, $\nu_{eq}\approx 0.087$). The figure also shows the far-from-equilibrium bistable region obtained when du/dt = dν/dt = 0 (black zone). The boundaries of this region are given by two saddle node (sn) lines meeting each other in a cusp. Inside this region, the system of ODEs admits two stable solutions and one unstable one, for the same control parameters. Each of these solutions is characterised by a certain amount of CO molecules and oxygen atoms on the surface. However, the unstable one is never observed in simulations.

**Figure 2:**
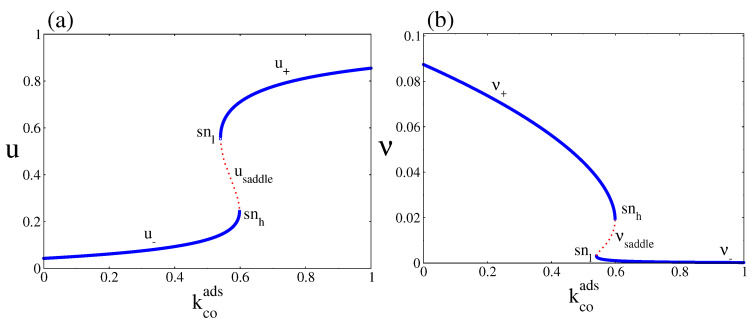
(**a**) and (**b**) Steady state bifurcation diagrams of the CO and oxygen coverages as a function of $k_{co}^{ads}$, for $k_{co}^{des}=0.15$. The figures clearly show a bistable region in which two NESS stable branches (blue lines) coexist with an unstable or saddle one (red dots). In all cases, the boundaries of the bistable region are characterised by saddle node bifurcations at the $sn_{l}$ and $sn_{h}$ points. Other parameters are $k_{o_{2}}^{ads}=0.2$, $k_{o}^{des}=20$, $k_{co_{2}}=0.5$, $k_{r}=50$, and ζ=4 (square lattice)

For clarity, Figure 2a and b show separately the steady state behaviour of *u* and ν as a function of $k_{co}^{ads}$, for $k_{co}^{des}=0.15$. Figure 2a shows a branch along which the CO coverage is small (denoted as $u_{-}$) and a branch along which the CO coverage is high (denoted as $u_{+}$). Figure 2b shows the corresponding bifurcation diagram for the oxygen coverage, in which we can also observe a lower branch denoted as $\nu_{-}$ and an upper branch denoted as $\nu_{+}$. The figures also show the existence of a region inside which the two NESS branches coexist with a middle one (red dots), which is always unstable (denoted as $u_{saddle}$ in the case of CO coverage and $\nu_{saddle}$ in the case of oxygen coverage). The boundaries of these bistable regions are characterised by the annihilation of the corresponding stable NESS branches with the saddle or unstable one (a saddle node bifurcation occurs at the $sn_{l}$ and $sn_{h}$ points) [23]. This unstable branch is not observed in simulations. The bistable region constitutes a so-called hysteresis loop. When $k_{co}^{ads}$ increases from zero, the system leads simultaneously along $u_{-}$ and $\nu_{+}$ until they disappear simultaneously in a saddle node bifurcation at the $sn_{h}$ point, then switches to $u_{+}$ and $\nu_{-}$, respectively. As $k_{co}^{ads}$ decreases from above, the system remains along $u_{+}$ and $\nu_{-}$ until it turns around the low $sn_{l}$ point and jumps to $u_{-}$ and $\nu_{+}$, respectively. Thus, the bistable region consists of the stable branches ($u_{-}$, $\nu_{+}$) and ($u_{+}$, $\nu_{-}$) coexisting with the unstable one given by ($u_{saddle}$, $\nu_{saddle}$).

**Figure 3:**
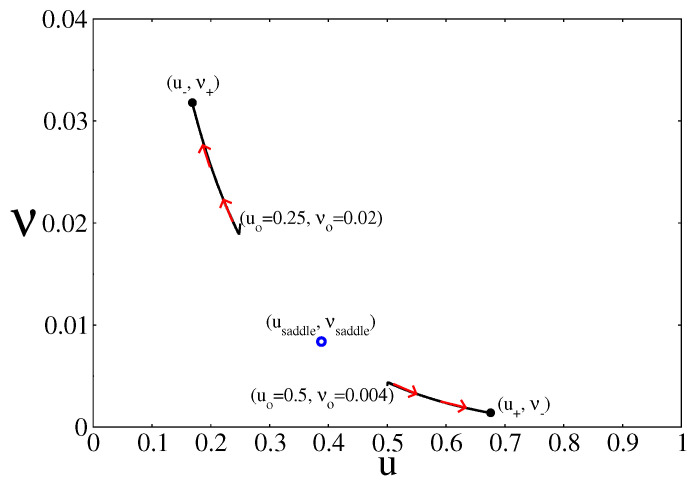
Time evolution of the system inside the bistable region and on the plane (*u*, ν), for two different initial conditions. In this case, $k_{co}^{ads}=0.57$ and $k_{co}^{des}=0.15$. Depending on the initial condition, the system converges to one of the two NESS branches of the bistable region. Other parameters are $k_{o_{2}}^{ads}=0.2$, $k_{o}^{des}=20$, $k_{co_{2}}=0.5$, $k_{r}=50$, and ζ=4 (square lattice)

The stability of the branches under weak perturbations is normally analysed in terms of the Jacobian matrix of Equations (19) and (20) evaluated at each of the steady state points along the branches [44]. The points along the branches ($u_{-}$, $\nu_{+}$) and ($u_{+}$, $\nu_{-}$) are stable, while the points along the saddle branch ($u_{saddle}$, $\nu_{saddle}$) are unstable. However, as Figure 3 shows, the selection between the two stable NESS branches depends on the initial condition of the simulation. As an example, the figure shows that starting from ($u_{o}=0.25$, $\nu_{o}=0.02$) leads to a point along the branch ($u_{-}$, $\nu_{+}$), while starting from ($u_{o}=0.5$, $\nu_{o}=0.004$) leds to a point along the branch ($u_{+}$, $\nu_{-}$). Outside the bistable region only one of the branches dominates no matter what the initial condition is.

**Figure 4:**
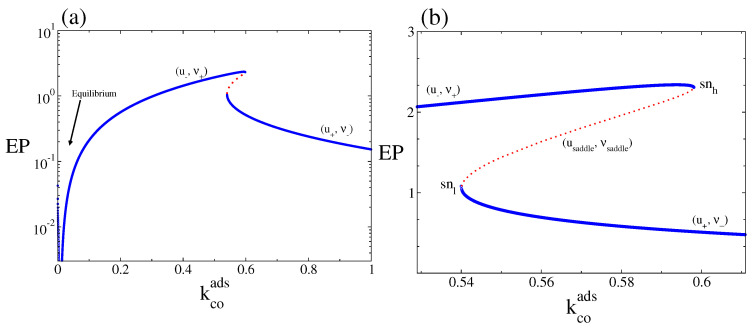
(**a**) Macroscopic entropy production rate, EP (Equation (22)), corresponding to the steady-state solutions of Equations (19) and (20) as a function of $k_{co}^{ads}$, for $k_{co}^{des}=0.15$. (**b**) The same EP but only for values of $k_{co}^{ads}$ around the bistable region. Full blue lines are the EP associated with the ($u_{-}$, $\nu_{+}$) and ($u_{+}$, $\nu_{-}$) stable branches and dotted line to the one of the unstable or saddle branch or ($u_{saddle}$, $\nu_{saddle}$). Other parameters are $k_{o_{2}}^{ads}=0.2$, $k_{o}^{des}=20$, $k_{co_{2}}=0.5$, $k_{r}=50$, and ζ=4 (square lattice).

We turn now to the properties of the macroscopic entropy production rate or dissipation. In Figure 4a the macroscopic entropy production rate, EP (Equation (22)), is plotted as a function of $k_{co}^{ads}$. We can observe the monostable and bistable behaviours associated with the branches presented in Figure 2. The entropy production vanishes at the thermodynamic equilibrium. As Figure 4 show, in the entire bistable interval, the dissipation along the points defined by the branch ($u_{-}$, $\nu_{+}$) is always larger than its value along the points associated with the branch ($u_{+}$, $\nu_{-}$). Thus, the state eventually selected for a given set of initial conditions is not necessarily the state where the system dissipates the most (which in the bistable region is the branch ($u_{-}$, $\nu_{+}$), for all values of $k_{co}^{ads}$).

### 4.2. Stochastic Analysis

The results presented above suggest that the macroscopic entropy production rate is not necessarily at its minimum or maximum value when the catalytic system is in a NESS. Depending on the initial conditions, the system can end up in a NESS with high or low entropy production rate. In this subsection, we explore whether this conclusion is still valid when the dynamics of the catalytic system are under the influence of the coverage fluctuations.

**Figure 5:**
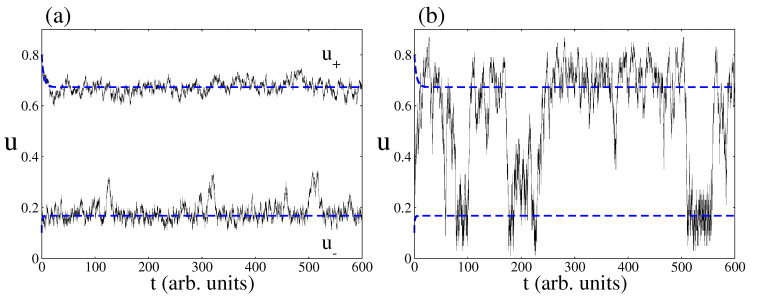
Time series of CO coverage from the deterministic and stochastic approaches. Blue dashed lines correspond to the deterministic prediction and full black lines to the stochastic one. (**a**) Stochastic simulations for a surface area of $N_{L}=1500$. Note as the stochastic trajectories follow on average the trajectories predicted by the deterministic approach; (**b**) Stochastic simulations with a surface area of $N_{L}=100$. Note the phenomenon of fluctuation-induced transitions. Other parameters are $k_{co}^{ads}=0.57$, $k_{co}^{des}=0.15$, $k_{o_{2}}^{ads}=0.2$, $k_{o}^{des}=20$, $k_{co_{2}}=0.5$, $k_{r}=50$, and ζ=4 (square lattice).

When decreasing the catalytic surface area, the predictions of the CME (Equation (9)) simulated by the Gillespie algorithm deviate from the predictions of the deterministic approach. One of the most prominent features is the phenomenon of fluctuations-induced transitions between the two non-equilibrium steady states of the bistable region [45]. As an example, Figure 5 shows time series pertaining to CO coverage inside the bistable region as obtained from both approaches, the deterministic and stochastic one. Blue dashed lines are the two non-equilibrium steady states predicted by the deterministic approach. As expected, depending on the initial condition, the catalytic system relaxes towards a NESS with a large amount of CO molecules on the surface or a NESS with a small number of CO molecules. In relation to the stochastic predictions, Figure 5a shows that, if the area of the surface is large enough, the stochastic time series follow on average the deterministic trajectories. However, Figure 5b shows that random transitions between the two non-equilibrium steady states may occur when the surface area is small enough.

**Figure 6:**
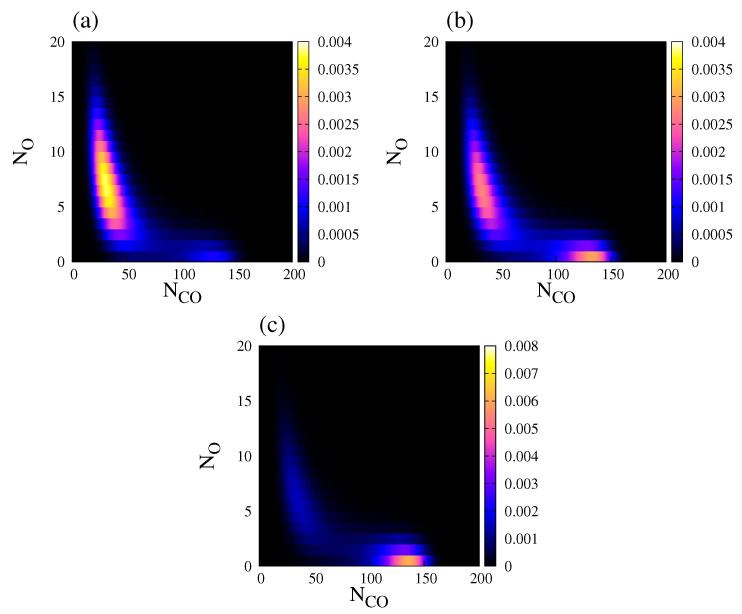
Normalised steady state probability distribution, $P_{st}\left(Z\right)$, of finding a population vector $Z=\{N_{CO},N_{O}\}$, for $N_{L}=200$. (**a**) $k_{co}^{ads}=0.55$; (**b**) $k_{co}^{ads}=0.56$; (**c**) $k_{co}^{ads}=0.57$. Other parameters are $k_{co}^{des}=0.15$, $k_{o_{2}}^{ads}=0.2$, $k_{o}^{des}=20$, $k_{co_{2}}=0.5$, $k_{r}=50$, and ζ=4 (square lattice). The steady state probability distribution was obtained after averaging over an ensemble (20 independent realisations sampled at a fixed time).

In a stochastic system, a useful quantity to study bistability is the normalised steady state probability distribution, $P_{st}\left(Z;t\right)$, of finding a population vector $Z=\{N_{CO},N_{O}\}$ [27]. Throughout this work, P(Z;t), is obtained by using the so-called Gillespie algorithm together with the transition rates presented in Table 1. Figure 6 shows the behaviour of $P_{st}\left(Z;t\right)$ projected on the plane ($N_{CO}$, $N_{O}$), for three different values of $k_{co}^{ads}$, and $k_{co}^{des}=0.15$. $P_{st}\left(Z;t\right)$ exhibits bimodal distribution in all three cases, but one of the peaks is usually much more dominant.

**Figure 7:**
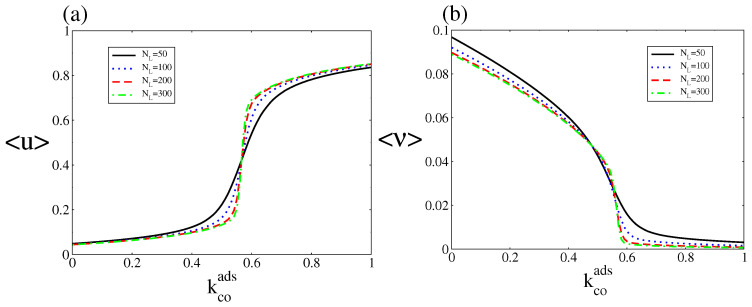
(**a**) and (**b**) Steady state bifurcation diagrams of the average CO and oxygen coverages as a function of $k_{co}^{ads}$, for $k_{co}^{des}=0.15$. Other parameters are $k_{o_{2}}^{ads}=0.2$, $k_{o}^{des}=20$, $k_{co_{2}}=0.5$, $k_{r}=50$, and ζ=4 (square lattice). The steady state probability distribution was obtained after averaging over an ensemble (20 independent realisations sampled at a fixed time). This figure should be compared with Figure 2.

The stationary probability distribution allows us to calculate the steady state average quantities

(27) $$\langle u\rangle =\sum_{Z}^{}uP_{st}\left(Z\right),$$

and

(28) $$\langle \nu \rangle =\sum_{Z}^{}\nu P_{st}\left(Z\right),$$

where *u*=$N_{CO}/N_{L}$ and *u*=$N_{O}/N_{L}$. Figure 7 shows 〈u〉, and 〈ν〉 versus $k_{co}^{ads}$ for different system sizes. The figure shows that the average oxygen coverage and CO coverage exhibit single-valued rather than the S-shaped multivalued behaviour of the deterministic approach. As in the case of equilibrium first-order phase transition, a transition from the steady state of low CO coverage and high oxygen coverage to the steady state of high CO coverage and low oxygen coverage occurs at a critical point, and it becomes sharper as $N_{L}$ increases. Hence, the state with low CO coverage and high oxygen coverage is selected below the critical point, while above this point the state with high CO coverage and low oxygen coverage is selected. Such kinetic phase transitions were first observed in the 80’s for a CO oxidation model that did not, however, obey microscopic reversibility (as it contained irreversible reactions) [46].

**Figure 8:**
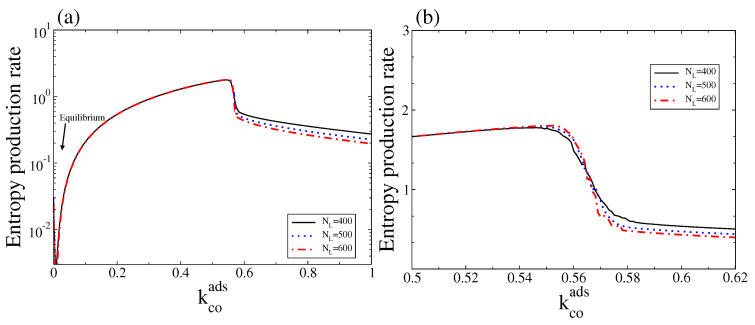
(**a**) Stochastic entropy production rate measured as $N_{L}^{-1}dS_{i}/dt$ versus $k_{co}^{ads}$ for three different system sizes, and $k_{co}^{des}=0.15$; (**b**) The same as in (**a**) but only for values of $k_{co}^{ads}$ around the bistable region. Other parameters are $k_{o_{2}}^{ads}=0.2$, $k_{o}^{des}=20$, $k_{co_{2}}=0.5$, $k_{r}=50$, and ζ=4 (square lattice). To calculate this entropy production rate, the steady state probability distribution was obtained after averaging over an ensemble (20 independent realisations sampled at a fixed time). This figure should be compared with Figure 4.

In Figure 8 we show the corresponding stochastic entropy production rate (Equation (15)) as a function of $k_{co}^{ads}$. As expected, the stochastic entropy production rate also vanishes at the thermodynamic equilibrium. Note also that, outside the bistable region, the stochastic entropy production rate follows the trend of the macroscopic entropy production rate, in particular close to equilibrium (for a comparison see Figure 4 and corresponding discussions). It also shows that, in contrast to the macroscopic entropy production rate, this quantity always follows a monostable behaviour along the bistable region (see Figure 4a,b for a comparison with the deterministic entropy production, EP). However, there is a critical point around which a transition from a state with high entropy production (associated with the branch with low CO and high oxygen coverage) to a state with low entropy production (associated with the branch with high CO and low oxygen coverage) occurs. Thus, a state of high (low) entropy production is selected below (above) the critical point.

## 5. Overall CO_2_ Production Rate

**Figure 9:**
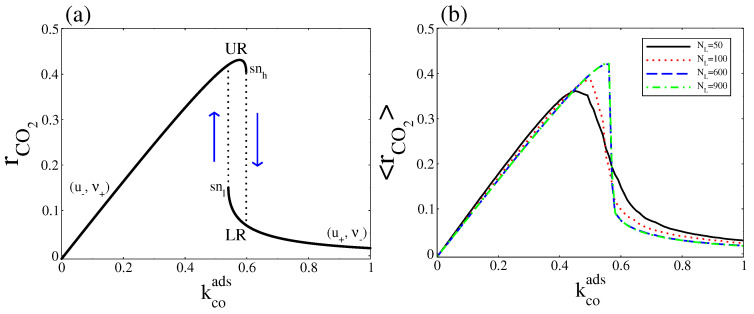
(**a**) Deterministic overall CO_2_ production rate; (**b**) Stochastic overall CO_2_ production rate at stationary state. In both cases, $k_{co}^{ads}$ was variated and $k_{co}^{des}$ was fixed at 0.15. Other parameters are $k_{o_{2}}^{ads}=0.2$, $k_{o}^{des}=20$, $k_{co_{2}}=0.5$, $k_{r}=50$, and ζ=4 (square lattice).

Finally, it is interesting to compare the overall CO production rate as calculated from the deterministic and stochastic approaches. For our deterministic mean-field description, with ζ=4, the deterministic overall reaction rate is given by (see Figure 9a)

(29) $$r_{CO_{2}}=w_{-3}-w_{+3}=k_{r}u\nu -2k_{co_{2}}{\left(1-u-\nu \right)}^{2}.$$

As Equation (29) shows, this expression can be zero, positive, or negative. When it is negative the CO${}_{2}$(gas) dissociative adsorption dominates, while when it is positive the CO(ads) + O(ads) reaction dominates. It is to equal zero only when the system is at thermodynamic equilibrium (in Figure 9a this occurs at $k_{co}^{ads}\approx 0.0075$). When the parameter $k_{co}^{ads}$ is varied, the two steady states define two separate stable branches that we call upper rate (UR) and lower rate (LR). The UR correspond to the points along the ($u_{-}$, $\nu_{+}$) branch, while the LR correspond to the points along the ($u_{+}$, $\nu_{-}$) branch. When $k_{co}^{ads}$ increases from zero, the reaction rate leads along the UR until it disappears in a saddle node bifurcation at the $sn_{h}$ point, then switches to LR. As $k_{co}^{ads}$ decreases from above, the reaction rate remains along LR until the branch turn around the $sn_{l}$ point and the reaction rate jumps to the UR (a hysteresis loop occurs). From Figure 2 and Figure 4, it is clear that, all along the bistable region, the UR has a higher entropy production rate.

The stochastic overall CO production rate is calculated using the Gillespie’s algorithm. We generate a reaction rate time series where each reading of the reaction rate is $R(t_{k})$ = $\left(r_{-3}-r_{+3}\right)/\left(N_{L}\Delta t\right)$, with Δt = $t_{k}-t_{k-1}=0.1$. The terms $r_{-3}$ and $r_{+3}$ are the numbers of elementary events of reaction CO(ads) + O(ads) and CO${}_{2}$(gas) dissociative adsorption, that occur during the time Δt, respectively. Then, to obtain $\langle r_{CO_{2}}\rangle$, we take the average over the number of readings. Instead of the hysteresis behaviour presented in Figure 9a, Figure 9b shows that, around a critical point, $\langle r_{CO_{2}}\rangle$ exhibits a monotonic transition from the UR to LR, which becomes sharper as the system size $N_{L}$ increases. Below the critical point the UR is selected, while above that point the LR is the one selected. These correspond respectively to the high and low entropy production rates calculated in Figure 8.

## 6. Summary and Conclusions

In this work, we explored the non-equilibrium thermodynamics and non-linear dynamics of a model for a catalytic surface reaction with emphasis on the question whether the entropy production rate is maximised, minimised, or does not achieve any extremum at a NESS. We considered a minimalistic model for the bistable catalytic CO oxidation reaction on single crystal surfaces, and explored the deterministic and stochastic aspects of it. From the deterministic approach, we derived the region of the control-parameter space where bistability is observed and we identified the equilibrium branch of the system. This phase diagram allows us to identify, inside the bistable region, a hysteresis loop in which a NESS characterised by a high CO${}_{2}$ production rate (reactive state) coexists with a NESS with a low CO${}_{2}$ production rate (less reactive state). We identified the characteristic S-shaped behaviour of bistability. The deterministic approach also predicts that, depending on the initial conditions, the system will reside in one of the two stable non-equilibrium states for an indefinite period of time.

The corresponding stochastic model exhibits, due to coverage fluctuations, random transitions between those non-equillibrium states. These transitions were analysed using the whole probability distribution of the catalytic system. We used this probability distribution to calculate the CO coverage and the oxygen coverage inside and outside the bistable region. We found that, instead of the deterministic S-shaped behaviour observed when $k_{co}^{ads}$ is varied, the stochastic framework predicts monotononic variations of these quantities. This indicates a first-order phase transition at a critical value of $k_{co}^{ads}=k$ which becomes sharper as the size of the system, $N_{L}$, increases. Hence, for $k_{co}^{ads}<k$ the high reactive state with a low CO coverage is selected, while for $k_{co}^{ads}>k$ the less reactive state with a high CO coverage is selected.

To analyse the non-equilibrium thermodynamic behaviour of the system, we calculated the macroscopic (deterministic) entropy production rate as well as its corresponding stochastic version. The macroscopic entropy production predicts that inside the bistable region, there are three entropy production rates, one for each steady state. Depending on the control parameters and initial conditions, the system can reach a stable state of low or high entropy production rate. Inside this bistable region, the state with a high entropy production rate corresponds to a NESS with a high CO_2_ production rate, while the state of low entropy production rate corresponds to a NEES characterised by a low CO_2_ production rate. In the stochastic description, there is a unique entropy production rate. Inside the bistable region, the stochastic entropy production rate exhibits a first-order phase transition at a critical point, which becomes sharper as $N_{L}$ increases. For $k_{co}^{ads}$ below the critical point (for $k_{co}^{ads}$ above the critical point), a state with high (low) entropy production is selected. These observations suggest that, in our model, the entropy production rate is neither maximised nor minimised for all non-equilibrium states.

It is important to emphasise that the results presented in this work only correspond to deterministic and average stochastic interpretations of entropy production. A more formal study will require an interpretation in terms of trajectory stochastic thermodynamics, information theory, and the recently established notion of stochastic least-action principle for dissipative systems [3,8,37,47]. This is an important and interesting extension of our work that will be explored in the near future. For example, one can wonder about the relation, as far as entropy production is concerned, between the deterministic trajectory and the set of trajectories around it associated with the presence of coverage fluctuations. In the present stochastic approach, these stochastic trajectories are averaged out. It should be also very convenient to extend this study to other type of dissipative structures observed in catalytic surface reaction systems, like for example oscillatory and excitable dynamics [48,49].

As a final note on the future extensions of this work, we would like to emphasise that, when dealing with non-equilibrium mesoscopic systems, we need to make a proper distinction between the predictions of the stochastic approach and those of the ODEs based on the traditional law of mass action kinetics. For example, it has been recognised that the size of the system, which does not appear in the ODEs, is an important bifurcation parameter [50,51]. In the context of catalytic surface reactions, a work along these lines was presented in [52]. Using an irreversible minimalistic model for the catalytic CO oxidation, a shift of the bistable region was predicted as a function of the size of the catalytic surface.

## Figures and Tables

**Table 1:** Processes, population changes, and transition rates for our well-mixed CME treatment of the dynamics of $Z=\{N_{CO},N_{O}\}$ for a surface with $N_{L}$ available sites. Parameters $k_{co}^{ads}$ and $k_{co}^{des}$ represent the rate constants for CO(gas) adsorption and CO(ads) desorption, respectively. $k_{o_{2}}^{ads}$ and $k_{o}^{des}$ are the rate constants for O${}_{2}$(gas) dissociative adsorption and O(ads) associative desorption. The parameter $k_{co_{2}}$ is the rate constant for CO${}_{2}$(gas) dissociative adsorption, and $k_{r}$ is the rate constant for CO(ads) + O(ads) reaction. ζ is the coordination number or the number of nearest neighbours of a site. In a 2D regular lattice ζ can take one of the following values: 3 for honeycomb-type lattice, 4 for a square lattice, and 6 for a hexagonal lattice [31,32]. Please note that the number of free sites on the surface is $N_{*}=N_{L}-N_{CO}-N_{O}$. In Appendix A, we demonstrate the consistency of these transition rates.

Process	Population Change	Transition Rate
CO(gas) adsorption	$\left(N_{CO},N_{O}\right)\to \left(N_{CO}+1,N_{O}\right)$	$W_{+1}=k_{co}^{ads}\left(N_{L}-N_{CO}-N_{O}\right)$
CO(ads) desorption	$\left(N_{CO},N_{O}\right)\to \left(N_{CO}-1,N_{O}\right)$	$W_{-1}=k_{co}^{des}N_{CO}$
O${}_{2}$(gas) dissociative adsorption	$\left(N_{CO},N_{O}\right)\to \left(N_{CO},N_{O}+2\right)$	$W_{+2}=\frac{\zeta k_{o_{2}}^{ads}}{2(N_{L}-1)}N_{*}\left(N_{*}-1\right)$
O(ads) associative desorption	$\left(N_{CO},N_{O}\right)\to \left(N_{CO},N_{O}-2\right)$	$W_{-2}=\frac{\zeta k_{o}^{des}}{2(N_{L}-1)}N_{O}\left(N_{O}-1\right)$
CO${}_{2}$(gas) dissociative adsorption	$\left(N_{CO},N_{O}\right)\to \left(N_{CO}+1,N_{O}+1\right)$	$W_{+3}=\frac{\zeta k_{co_{2}}}{2(N_{L}-1)}N_{*}\left(N_{*}-1\right)$
CO(ads) + O(ads) reaction	$\left(N_{CO},N_{O}\right)\to \left(N_{CO}-1,N_{O}-1\right)$	$W_{-3}=\frac{\zeta k_{r}}{N_{L}-1}N_{CO}N_{O}$

**Table 2:** Reaction rates or fluxes pertaining to Equations (19) and (20). Please note that the macroscopic rate constants are $K_{co}^{ads}=k_{co}^{ads}$, $K_{co}^{des}=k_{co}^{des}$, $K_{o_{2}}^{ads}=\zeta k_{o_{2}}^{ads}/2$, $K_{o}^{des}=\zeta k_{o}^{des}/2$, $K_{co_{2}}=\zeta k_{co_{2}}/2$, and $K_{r}=\zeta k_{r}$.

Process	Reaction Rates or Fluxes
CO(gas) adsorption	$w_{+1}=K_{co}^{ads}\left(1-u-\nu \right)$
CO(ads) desorption	$w_{-1}=K_{co}^{des}u$
O${}_{2}$(gas) dissociative adsorption	$w_{+2}=2K_{o_{2}}^{ads}{\left(1-u-\nu \right)}^{2}$
O(ads) associative desorption	$w_{-2}=2K_{o}^{des}\nu^{2}$
CO${}_{2}$(gas) dissociative adsorption	$w_{+3}=K_{co_{2}}{\left(1-u-\nu \right)}^{2}$
CO(ads) + O(ads) reaction	$w_{-3}=K_{r}u\nu$

## Abbreviations

The following abbreviations are used in this manuscript:

NESS	Non-equilibrium steady state
ODEs	Ordinary differential equations
CO	Carbon monoxide
CO_2_	Carbon dioxide
O_2_	Oxygen
LH	Langmuir-Hinshelwood
CME	Chemical master equations
2D	two dimensional
EP	Macroscopic entropy production rate
sn	Saddle node
UR	Upper rate
LR	Lower rate
MinEPP	Minimium entropy production principle
MaxEPP	Maximium entropy production principle

## Appendix A. Transition Rates and Detailed Balance

Here, we show that the transition rates, $W_{\sigma }$, satisfy the following constraint [49]

(A1) $$\frac{W_{-\sigma }\left(Z\right)}{W_{\sigma }\left(Z-\omega_{\sigma }\right)}=\frac{P_{eq}\left(Z-\omega_{\sigma }\right)}{P_{eq}\left(Z\right)},$$

with

(A2) $$P_{eq}\left(Z\right)=\frac{N_{L}!}{N_{CO}!N_{O}!N_{*}!}u_{eq}^{N_{CO}}\nu_{eq}^{N_{O}}{\left(1-u_{eq}-\nu_{eq}\right)}^{N_{*}},$$

where $N_{*}=N_{L}-N_{CO}-N_{O}$. It is easy to show that ${\langle N_{CO}\rangle }_{eq}=N_{L}u_{eq}$, ${\langle N_{O}\rangle }_{eq}=N_{L}\nu_{eq}$, and ${\langle N_{*}\rangle }_{eq}=\left(N_{L}-{\langle N_{CO}\rangle }_{eq}-{\langle N_{O}\rangle }_{eq}\right)=N_{L}\left(1-u_{eq}-\nu_{eq}\right)$. For simplicity, we omitted the arrow used in Equation (10). As before, in $Z=\{N_{CO},N_{O}\}$, $N_{CO}$ is the number of adsorbed CO species on the surface and $N_{O}$ is the number of adsorbed oxygen atoms. The stoichiometric vectors for forward reactions are $\omega_{+1}=\left\{1,0\right\}$, $\omega_{+2}=\left\{0,2\right\}$, and $\omega_{+3}=\left\{1,1\right\}$, and the once for the reversed reactions $\omega_{-1}=-\omega_{+1}$, $\omega_{-2}=-\omega_{+2}$, and $\omega_{-3}=-\omega_{+3}$.

### Appendix A.1. CO(gas) Adsorption and CO(ads) Desorption.

For adsorption (forward step) $\omega_{+1}=\left\{1,0\right\}$ and for desorption (reverse step) $\omega_{-1}=\left\{-1,0\right\}$. Then, the constraint leads to

(A3) $$\frac{W_{-1}\left(N_{CO},N_{O}\right)}{W_{+1}\left(N_{CO}-1,N_{O}\right)}=\frac{P_{eq}\left(N_{CO}-1,N_{O}\right)}{P_{eq}\left(N_{CO},N_{O}\right)}.$$

After replacing $P_{eq}\left(N_{CO},N_{O}\right)$, one gets

(A4) $$\frac{W_{-1}\left(N_{CO},N_{O}\right)}{W_{+1}\left(N_{CO}-1,N_{O}\right)}=\frac{1-u_{eq}-\nu_{eq}}{u_{eq}}\frac{N_{CO}}{N_{*}+1},$$

where $N_{*}=N_{L}-N_{CO}-N_{O}$. At equilibrium, $w_{+1}=w_{-1}$ (see Table 2), and

(A5) $$\frac{\left(1-u_{eq}-\nu_{eq}\right)}{u_{eq}}=\frac{K_{co}^{des}}{K_{co}^{ads}}.$$

Then

(A6) $$\frac{W_{-1}\left(N_{CO},N_{O}\right)}{W_{+1}\left(N_{CO}-1,N_{O}\right)}=\frac{K_{co}^{des}}{K_{co}^{ads}}\frac{N_{CO}}{N_{*}+1}=K_{eq}^{co}\frac{N_{CO}}{N_{*}+1},$$

where $K_{eq}^{co}$ is the equilibrium contant. Finally, we conclude that

(A7) $$W_{+1}\left(N_{CO},N_{O}\right)=k_{co}^{ads}\left(N_{L}-N_{CO}-N_{O}\right)=k_{co}^{ads}N_{*},$$

and

(A8) $$W_{-1}\left(N_{CO},N_{O}\right)=k_{co}^{des}N_{CO}.$$

satisfy Equation (A6), where $K_{eq}^{co}=K_{co}^{des}/K_{co}^{ads}$, $K_{co}^{des}=k_{co}^{des}$, and $K_{co}^{ads}=k_{co}^{ads}$

### Appendix A.2. Dissociative O_2_(gas) Adsorption and Associative O(ads) Desorption.

For adsorption (forward step) $\omega_{+2}=\left\{0,2\right\}$ and for desorption (reverse step) $\omega_{-2}=\left\{0,-2\right\}$. Then, the constraint is

(A9) $$\frac{W_{-2}\left(N_{CO},N_{O}\right)}{W_{+2}\left(N_{CO},N_{O}-2\right)}=\frac{P_{eq}\left(N_{CO},N_{O}-2\right)}{P_{eq}\left(N_{CO},N_{O}\right)}.$$

After replacing $P_{eq}\left(N_{CO},N_{O}\right)$, one gets

(A10) $$\frac{W_{-2}\left(N_{CO},N_{O}\right)}{W_{+2}\left(N_{CO},N_{O}-2\right)}=\frac{{\left(1-u_{eq}-\nu_{eq}\right)}^{2}}{\nu_{eq}^{2}}\frac{N_{O}\left(N_{O}-1\right)}{\left(N_{*}+2\right)\left(N_{*}+1\right)},$$

where $N_{*}=N_{L}-N_{CO}-N_{O}$. At equilibrium, $w_{+2}=w_{-2}$ (see Table 2), and

(A11) $$\frac{{\left(1-u_{eq}-\nu_{eq}\right)}^{2}}{\nu_{eq}^{2}}=\frac{K_{o}^{des}}{K_{o_{2}}^{ads}}.$$

Then

(A12) $$\frac{W_{-2}\left(N_{CO},N_{O}\right)}{W_{+2}\left(N_{CO},N_{O}-2\right)}=\frac{K_{o}^{des}}{K_{o_{2}}^{ads}}\frac{N_{O}\left(N_{O}-1\right)}{\left(N_{*}+2\right)\left(N_{*}+1\right)}=K_{eq}^{o}\frac{N_{O}\left(N_{O}-1\right)}{\left(N_{*}+2\right)\left(N_{*}+1\right)},$$

where $K_{eq}^{o}$ is the equilibrium contant. Finally, we conclude that

(A13) $$W_{+2}\left(N_{CO},N_{O}\right)=\frac{\zeta k_{o_{2}}^{ads}}{2(N_{L}-1)}N_{*}\left(N_{*}-1\right),$$

and

(A14) $$W_{-2}\left(N_{CO},N_{O}\right)=\frac{\zeta k_{o}^{des}}{2(N_{L}-1)}N_{O}\left(N_{O}-1\right).$$

satisfy Equation (A12), where $K_{eq}^{o}=K_{o}^{des}/K_{o_{2}}^{ads}$, $K_{o_{2}}^{ads}=zk_{o_{2}}^{ads}/2$, and $K_{o}^{des}=zk_{o}^{des}/2$.

### Appendix A.3. CO(ads)+O(ads) Reaction and CO_2_(gas) Dissociative Adsorption

For adsorption (forward step) $\omega_{+3}=\left\{+1,+1\right\}$ and for desorption after reaction (reverse step) $\omega_{-3}=\left\{-1,-1\right\}$. Then, the constraint is

(A15) $$\frac{W_{-3}\left(N_{CO},N_{O}\right)}{W_{+3}\left(N_{CO}-1,N_{O}-1\right)}=\frac{P_{eq}\left(N_{CO}-1,N_{O}-1\right)}{P_{eq}\left(N_{CO},N_{O}\right)}.$$

After replacing $P_{eq}\left(N_{CO},N_{O}\right)$, one gets

(A16) $$\frac{W_{-3}\left(N_{CO},N_{O}\right)}{W_{+3}\left(N_{CO}-1,N_{O}-1\right)}=\frac{{\left(1-u_{eq}-\nu_{eq}\right)}^{2}}{u_{eq}\nu_{eq}}\frac{N_{CO}N_{O}}{\left(N_{*}+2\right)\left(N_{*}+1\right)},$$

where $N_{*}=N_{L}-N_{CO}-N_{O}$. At equilibrium, $w_{+3}=w_{-3}$ (see Table 2), and

(A17) $$\frac{{\left(1-u_{eq}-\nu_{eq}\right)}^{2}}{u_{eq}\nu_{eq}}=\frac{K_{r}}{K_{co_{2}}}.$$

Then

(A18) $$\frac{W_{-3}\left(N_{CO},N_{O}\right)}{W_{+3}\left(N_{CO}-1,N_{O}-1\right)}=\frac{K_{r}}{K_{co_{2}}}\frac{N_{CO}N_{O}}{\left(N_{*}+2\right)\left(N_{*}+1\right)}=K_{eq}^{r}\frac{N_{CO}N_{O}}{\left(N_{*}+2\right)\left(N_{*}+1\right)},$$

where $K_{eq}^{r}$ is the equilibrium contant. Finally, it is easy to check that

(A19) $$W_{+3}\left(N_{CO},N_{O}\right)=\frac{\zeta k_{co_{2}}}{2(N_{L}-1)}N_{*}\left(N_{*}-1\right),$$

and

(A20) $$W_{-3}\left(N_{CO},N_{O}\right)=\frac{\zeta k_{r}}{N_{L}-1}N_{CO}N_{O}.$$

satisfy Equation (A18), where $K_{eq}^{r}=K_{r}/K_{co_{2}}$, $K_{co_{2}}=zk_{co_{2}}/2$, and $K_{r}=zk_{r}$.

Finally, note that chemical equilibrium implies

(A21) $$\frac{K_{eq}^{co}{\left(K_{eq}^{o}\right)}^{1/2}}{K_{eq}^{r}}=1.$$
